# Osteoporose bei pneumologischen Erkrankungen: Gemeinsame Leitlinie der Österreichischen Gesellschaft für Knochen und Mineralstoffwechsel (ÖGKM) und der Österreichischen Gesellschaft für Pneumologie (ÖGP)

**DOI:** 10.1007/s00508-021-01896-x

**Published:** 2021-06-16

**Authors:** Christian Muschitz, Ralf Harun Zwick, Judith Haschka, Hans Peter Dimai, Martina Rauner, Karin Amrein, Robert Wakolbinger, Peter Jaksch, Ernst Eber, Peter Pietschmann

**Affiliations:** 1 Medical University of Vienna (external lecturer), Waehringer Guertel 18–20, 1090 Wien, Österreich; 2 Medical Department II – VINFORCE, St. Vincent Hospital Vienna (Barmherzige Schwestern Krankenhaus Wien), Stumpergasse 13, 1060 Wien, Österreich; 3 Therme Wien Med, Kurbadstraße 14, 1100 Wien, Österreich; 4 1st Medical Department at Hanusch Hospital, Ludwig Boltzmann Institute of Osteology, Hanusch Hospital of the WGKK and AUVA Trauma Center, 1140 Wien, Österreich; 5 Karl Landsteiner Institute for Rheumatology and Gastroenterology, Rheuma-Zentrum Wien-Oberlaa, 1100 Wien, Österreich; 6 Division of Endocrinology and Diabetology, Medical University of Graz, Auenbruggerplatz 15, 8036 Graz, Österreich; 7 Divisions of Endocrinology and Molecular Bone Biology, Department of Medicine III, Medical Center, Technical University Dresden, 01307 Dresden, Deutschland; 8 Division of Endocrinology and Diabetology, Medical University of Graz, Auenbruggerplatz 15, 8036 Graz, Österreich; 9 Department of Physical and Rehabilitation Medicine (PRM), Clinic Donaustadt, Academic Teaching Hospital of the Medical University of Vienna, Langobardenstraße 122, 1220 Wien, Österreich; 10 Department of Thoracic Surgery, Medical University of Vienna, Waehringer Guertel 18–20, 1090 Wien, Österreich; 11 Division of Paediatric Pulmonology and Allergology, Department of Paediatrics and Adolescent Medicine, Medical University of Graz, Auenbruggerplatz 34/2, Graz, 8036 Österreich; 12 Department of Pathophysiology and Allergy Research, Center of Pathophysiology, Infectiology and Immunology, Medical University of Vienna, Waehringer Guertel 18–20, 1090 Wien, Österreich

**Keywords:** Osteoporose, Lungenerkrankungen, Leitlinie, Frakturrisiko, Diagnostik, Osteoporosis, Pulmonary diseases, Guideline, Fracture risk, Diagnosis

## Abstract

Asthma und COPD sind die häufigsten obstruktiven Atemwegserkrankungen. Die chronische Inflammation bedingt eine Induktion von proinflammatorischen Zytokinkaskaden. Neben der systemischen Inflammation tragen Hypoxämie, Hyperkapnie, eine katabole Stoffwechsellage, eine gonadale oder eine Schilddrüsendysfunktion, eine muskuloskelettale Dysfunktion und Inaktivität sowie Vitamin D‑Mangel zu einem erhöhten Knochenbruchrisiko bei. Iatrogene Ursachen der Osteoporose sind die zum Teil langjährigen Anwendungen von inhalativen oder systemischen Glukokortikoiden (GC). Die inhalative GC Applikation bei Asthma ist oft schon im Kindes- und Jugendalter indiziert, aber auch interstitielle Lungenerkrankungen wie die chronisch organisierende Pneumonie, die Sarkoidose oder rheumatische Erkrankungen mit Lungenbeteiligung werden mit inhalativen oder oralen GC behandelt. Bei PatientInnen mit zystischer Fibrose kommt es durch die Malabsorption im Rahmen der Pankreasinsuffizienz, durch Hypogonadismus und chronische Inflammation mit erhöhter Knochenresorption zu einer Abnahme der Knochenstruktur. Nach Lungentransplantation ist die Immunsuppression mit GC ein Risikofaktor.

Die pneumologischen Grunderkrankungen führen zu einer Veränderung der trabekulären und kortikalen Mikroarchitektur des Knochens und zu einer Verminderung von osteologischen Formations- und Resorptionsmarkern. Hyperkapnie, Azidose und Vitamin D‑Mangel können diesen Prozess beschleunigen und somit das individuelle Risiko für osteoporotische Fragilitätsfrakturen erhöhen.

Eine Knochendichtemessung mit einem T‑Score < −2,5 ist ein Schwellenwert zur Diagnose der Osteoporose, die überwiegende Mehrzahl aller osteoporotischen Frakturen tritt bei einem T‑Score von > −2,5 auf. Eine niedrig-traumatische Fraktur in der Anamnese indiziert eine osteologische Therapie.

Neben der Optimierung des Vitamin D‑Spiegels sind sämtliche in Österreich zur Behandlung der Osteoporose zugelassenen antiresorptiv oder anabol wirksamen Medikamente auch bei pneumologischen PatientInnen mit einem erhöhten Knochenbruchrisiko entsprechend der nationalen Erstattungskriterien indiziert.

## Abstract (en)

Chronic inflammation induces proinflammatory cytokine cascades. In addition to systemic inflammation, hypoxemia, hypercapnia, a catabolic metabolism, gonadal or thyroid dysfunction, musculoskeletal dysfunction and inactivity as well as vitamin D deficiency contribute to an increased risk of fragility fractures. Iatrogenic causes of osteoporosis are long-term use of inhaled or systemic glucocorticoids (GC). Inhalative GC application in asthma is often indicated in childhood and adolescence, but interstitial lung diseases such as chronic organizing pneumonia, COPD, sarcoid or rheumatic diseases with lung involvement are also treated with inhalative or oral GC. In patients with cystic fibrosis, malabsorption in the context of pancreatic insufficiency, hypogonadism and chronic inflammation with increased bone resorption lead to a decrease in bone structure. After lung transplantation, immunosuppression with GC is a risk factor.

The underlying pneumological diseases lead to a change in the trabecular and cortical bone microarchitecture and to a reduction in osteological formation and resorption markers. Hypercapnia, acidosis and vitamin D deficiency can accelerate this process and thus increase the individual risk of osteoporotic fragility fractures.

A bone mineral density measurement with a T‑Score < –2.5 is a threshold value for the diagnosis of osteoporosis; in contrast the vast majority of all osteoporotic fractures occur with a T‑Score > –2.5. A history of low-trauma fracture indicates osteological therapy.

All antiresorptive or anabolic drugs approved in Austria for the treatment of osteoporosis are also indicated for pneumological patients with an increased fragility fracture risk of bone fractures in accordance with the national reimbursement criteria.

## Einleitung

Basierend auf den Ergebnissen einer Consensus Development Conference im Jahr 1991 ist die Osteoporose gegenwärtig als eine Skelett-Erkrankung definiert, welche durch eine verminderte Knochenmasse sowie eine mikroarchitektonische Störung des Knochengewebes charakterisiert ist, mit der Folge eines erhöhten Frakturrisikos (Fragilitätsfrakturen, niedrig-traumatische Frakturen). Diese Definition wurde im Jahr 1994 durch eine WHO-Arbeitsgruppe um eine operationale Definition erweitert, welche zunächst nur für die postmenopausale Osteoporose, später aber auch für die Osteoporose des Mannes etabliert wurde [1].

Diese operationale Definition basiert auf einer Messung der Knochenmineraldichte (KMD) mittels Dual Energy X‑ray Absorptiometry (DXA), dessen Ergebnis als T‑Score ausgedrückt wird. Der T‑Score ist die Standardabweichung eines individuell gemessenen Knochenmineraldichte-Wertes vom mittleren Normwert knochengesunder, junger Erwachsener. Liegt der T‑Score bei −2,5 oder darunter, liegt definitionsgemäß eine Osteoporose vor. Zu berücksichtigen ist, dass dieser ursprünglich rein zur Diagnosestellung etablierte Schwellenwert sehr häufig auch als Therapie-Schwellenwert eingesetzt wird, obwohl die überwiegende Mehrzahl aller osteoporotischen Frakturen bei einem T‑Score von > −2,5 auftritt [2].

Der Verlust an KMD ist mit zunehmendem Lebensalter eine unausweichliche Konsequenz. In der weiblichen Population beginnt dieser kurz vor Beginn der Menopause, ist perimenopausal beschleunigt und setzt sich in weiterer Folge fort. Letzteres gilt auch mit einer zeitlichen Verzögerung für die männliche Population [3].

Die sekundären Ursachen der Osteoporose umfassen eine heterogene Vielfalt an Erkrankungen, Medikamenten oder exogenen Noxen. Es ist wichtig, in der klinischen Routine derartige Risikofaktoren frühzeitig zu erkennen und das individuelle Knochenbruchrisiko zu bestimmen. Klinische Risikofaktoren tragen zusätzlich zu einer erhöhten Knochenfragilität und einer verminderten Knochenqualität bei, sie sind häufig stärker als der altersabhängige Verlust einzustufen. Bei entsprechender Risikostratifizierung und frühzeitiger spezifischer osteologischer Behandlung sind derartige Veränderungen in der Regel reversibel [4].

Im Kontext von Lungenerkrankungen ist die chronisch obstruktive Lungenerkrankung (COPD) ein klassisches Beispiel mit erhöhten proinflammatorischen Zytokinen (vorrangig Tumor Nekrose Faktor alpha, TNF-α) als Trigger Faktor für die Entstehung von Osteoporose, kardiovaskulären Erkrankungen oder Diabetes. Neben Inflammation und/oder systemischen Glukokortikoiden tragen ein Vitamin D‑Mangel, eine verminderte Muskelmasse und Muskelkraft, Immobilisierung, Rauchen, Veränderungen des Body Mass Index (BMI), Hypogonadismus oder verminderte Spiegel von insulin-like growth factor‑1 (IGF-1) zur Erhöhung des individuellen Knochenbruchrisikos (sekundäre Osteoporose) bei [5, 6]. Durch den langjährigen katabolen Knochenstoffwechsel mit progredientem Verlust der skelettalen Integrität in Abhängigkeit vom Schweregrad der Grunderkrankung stellen vertebrale Frakturen in dieser PatientInnen Population ein häufiges und nachhaltiges Problem dar. PatientInnen mit Wirbelkörperfrakturen zeigen zudem eine herabgesetzte Lungenfunktion mit reduziertem forciertem exspiratorischen Volumen und reduzierter Vitalkapazität [7, 8].

## Pneumologische Erkrankungen und Osteoporose

### Obstruktive Lungenerkrankungen

Asthma und COPD sind die häufigsten obstruktiven Lungenerkrankungen; es finden sich Unterschiede in Pathogenese und Klinik, aber auch Gemeinsamkeiten in Hinblick auf die Entstehung einer Osteoporose (Tab. 1).

**Table 1:** 

Merkmal	COPD	Asthma
Alter bei Erstdiagnose	Meist nicht vor der 6. Lebensdekade	Häufig: Kindheit, Jugend
Tabakrauchen	Direkter Kausalzusammenhang	Kein direkter Kausalzusammenhang; Verschlechterung durch Tabakrauchen möglich
Hauptbeschwerden	Atemnot bei Belastung	Anfallsartig auftretende Atemnot
Verlauf	Meist progredient	Variabel, episodisch
Allergie	Kein direkter Kausalzusammenhang	Häufig
Obstruktion	Immer nachweisbar	Variabel, reversibel, oft aktuell nicht vorhanden
Diffusionskapazität	Oft erniedrigt	Meist normal
FeNO	Normal bis niedrig	Oft erhöht
Bluteosinophile	Meist normal	Häufig erhöht
Reversibilität der Obstruktion	Nie voll reversibel	Diagnostisches Kriterium, wenn voll reversibel
Überempfindlichkeit der Atemwege	Selten	Meist vorhanden
Ansprechen der Obstruktion auf Kortikosteroide	Selten	Regelhaft vorhanden

Bei beiden Erkrankungen liegt eine chronische Inflammation vor, aber es spielen viele weitere Faktoren eine relevante Rolle (vgl. Kapitel Pathophysiologie der Osteoporose bei COPD). Während die Genese bei der COPD durch die Raucheranamnese häufig rasch beantwortet werden kann, unterscheiden wir beim Asthma unterschiedliche Phänotypen, wie zum Beispiel allergisches und nicht allergisches Asthma oder das Adipositas assoziierte Asthma bronchiale, wo Adipokine und andere Zytokinkaskaden eine ähnliche Rolle wie bei Diabetes spielen dürften.

In der Differenzialdiagnose beider Erkrankungen spielt das Ansprechen der Atemwegsobstruktion auf Kortikosteroide eine entscheidende Rolle, bei Asthma ist dies regelhaft vorhanden, bei COPD nicht (Tab. 1).

#### COPD

Bei COPD liegt laut einem rezenten Reviews die Prävalenz einer Osteoporose global bei 38 % (95 % CI, 34–43) [10]. Das Vorliegen der COPD erhöht die Wahrscheinlichkeit auf Osteoporose erheblich (OR, 2,83). Neben der systemischen Inflammation fördert eine multifaktorielle Kaskade durch Hypoxämie, Hyperkapnie, katabole Stoffwechsellage, muskuloskelettale Dysfunktion und Inaktivität die Osteoporose maßgeblich. Bei älteren PatientInnen mit COPD liegt zudem eine gonadale- oder Schilddrüsendysfunktion vor, weiters besteht häufig ein Vitamin D3-Mangel häufig anzutreffen. Laut der Meta-Analyse von Chen et al. sind ein BMI < 18,5 (OR, 4,26) und das Vorliegen einer Sarkopenie (OR, 3,65) die wichtigsten Risikofaktoren [10].

##### COPD Therapie: GOLD Leitlinien 2021

**Figure 1:**
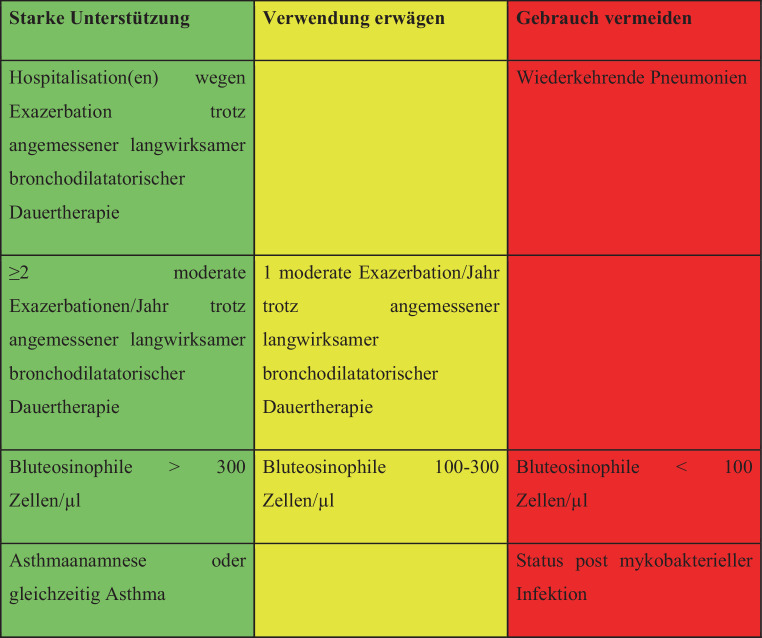

Inhalative Kortikosteroide (ICS) spielen in der Therapie der COPD eine untergeordnete Rolle (siehe www.goldcopd.org). Im Vordergrund stehen Bronchodilatatoren in Form von Anticholinergika und β2-Sympathomimetika, die Gabe von ICS ist nur bei Eosinophilie (> 300/μl), Asthmaanamnese oder gehäuften Exazerbationen innerhalb der letzten 12 Monate indiziert (Abb. 1). Die langfristige Gabe von oralen Kortikosteroiden (OCS) wird grundsätzlich nicht empfohlen, da die Nebenwirkungen den Benefit überwiegen (Evidenz A).

#### ASTHMA

ICS gelten als Basistherapie, werden bereits in der Kindheit eingesetzt und somit oft über Jahrzehnte inhaliert. Zu betonen ist, dass ICS die effektivste antiinflammatorische Therapie darstellen und somit unverzichtbar sind, da sie auch zu einer Reduktion von Exazerbationen und somit der Einnahme von OCS beitragen.

Die Rolle der ICS in Bezug auf eine Osteoporose wird bei Asthma seit langer Zeit kontrovers diskutiert [11]. Eine rezente Publikation aus einer englischen Datenbank mit > 650.000 PatientInnen, in der 138.000 Asthmatiker mit 520.000 Nicht-Asthmatikern verglichen wurden, zeigte ein höheres Osteoporoserisiko (adjusted Hazard Ratio-aHR 1,18, 95 % CI 1,13–1,23) und Frakturrisiko (aHR 1,12, 95 % CI 1,07–1,16) in der Asthmakohorte. Dabei war eine Dosis/Wirkungsbeziehung zwischen der Einnahme von OCS und Osteoporose zu sehen [12].

##### Asthmatherapie: GINA Leitlinien 2020

Die internationalen Asthmaleitlinien wurden im Dezember 2020 neu überarbeitet; die Rolle der ICS bleibt unverändert, die Einnahme von OCS ist erst in der Stufe 5 vorgesehen [13]. Hier haben sich in den letzten Jahren potente Medikamente wie Anti IGE, Anti IL5/5R oder Anti IL4R Antagonisten durchgesetzt. OCS bleiben in niedriger Dosierung unter Berücksichtigung der Nebenwirkungen eine Alternative als ultima ratio. In den neuen Global Initiative for Asthma (GINA) Guidelines wird angeführt, dass die langfristige Einnahme von hochdosierten ICS zu einem erhöhten Osteoporoserisiko führt. Asthmatiker mit einer Einnahme von niedrig dosiertem oralen Kortikosteroid (< 7,5 mg Prednisolonäquivalent) sollten über die Nebenwirkungen aufgeklärt werden und auf das Vorliegen einer Osteoporose gescreent werden. Bei einer hochdosierten systemischen Therapie mit > 7,5 mg Prednisolonäquivalent > 3 Monate wird eine antiresorptive Therapie empfohlen.

### Interstitielle Lungenerkrankungen

Bei interstitiellen Lungenerkrankungen wie der chronisch organisierenden Pneumonie, Sarkoidose oder rheumatischen Erkrankungen mit Lungenbeteiligung werden häufig ICS oder OCS über mehrere Monate verordnet.

In der Therapie der Lungenfibrose spielt die Therapie mit ICS oder OCS keine Rolle, dennoch ist aufgrund der systemischen Inflammation von einer hohen Osteoporosewahrscheinlichkeit auszugehen. Die rezenten deutsch/österreichischen Leitlinien zur Diagnostik der Lungenfibrose und auch die letzten ATS/ERS Leitlinien gehen jedoch nicht darauf ein [14, 15].

### Zystische Fibrose

Bei PatientInnen mit zystischer Fibrose beginnt die Entwicklung einer Osteoporose häufig schon in der Adoleszenz, mit Untergewicht und schlechter Lungenfunktion als starken Prädiktoren. Es kommt durch die Malabsorption im Rahmen der exokrinen Pankreasinsuffizienz, durch Hypogonadismus und chronische Inflammation mit erhöhter Knochenresorption zu einer Abnahme der Knochenstruktur [16]. Das Ausmaß des erhöhten Frakturrisikos ist altersäbhängig und hängt zu einem großen Teil von anderen prävalenten Risikofaktoren, wie vorangegangene niedrig-traumatische Fraktur, oder Vorbereitung zur oder Zustand nach Lungentransplantation ab [17].

## Pathophysiologie der Osteoporose bei COPD

Obwohl in der Literatur ein sehr klarer Zusammenhang zwischen COPD und Osteoporose beschrieben wird, sind die dieser Assoziation zugrundeliegenden pathophysiologischen Mechanismen nur unzureichend erforscht [10]. Analysen der Knochenmikrostruktur bei Patientinnen mit COPD haben eine Verminderung der Trabekelzahl, der Trabekeldicke und eine Verminderung der trabekulären Konnektivität ergeben; im Bereich der Kortikalis fand sich eine Verminderung der Dicke und eine erhöhte kortikale Porosität [18]. In dieser Studie wurde mit Hilfe der dynamischen Histomorphometrie gezeigt, dass die COPD-Patientinnen eine Verminderung der Knochenformation aufweisen. Diese Ergebnisse stehen mit einer Studie, welche Knochenumbau-Marker bei COPD bestimmt und eine Verminderung von Formations- und Resorptionsmarkern gefunden hat, im Einklang [19]. Tsukamoto et al. haben ein vielversprechendes Maus-Modell für die COPD beschrieben; hier finden sich Veränderungen der trabekulären Mikrostruktur, Hinweise auf eine verminderte Knochenformation und eine Atrophie der Typ 1‑Muskelfasern [20].

In der Pathophysiologie der COPD spielen Entzündungsprozesse eine zentrale Rolle; viele proinflammatorische Zytokine greifen auch in den Knochenstoffwechsel ein [21–23]. Bon et al. analysierten bei PatientInnen mit COPD den Zusammenhang zwischen Entzündungsparametern und dem Knochenumbau und fanden beispielsweise eine positive Korrelation zwischen TNF‑α und den sogenannten Crosslaps (CTX, Knochenabbaumarker) und dem Prokollagen-Typ I‑N-Propeptid (PINP, Knochenanbaumarker) [24]. Diese Ergebnisse deuten darauf hin, dass hohe Entzündungsaktivität bei COPD den Knochenstoffwechsel negativ beeinflussen. Eine wichtige Rolle in der Pathogenese der Osteoporose bei COPD spielt auch die anti-inflammatorische Therapie mit Glukokortikoiden.

**Figure 2:**
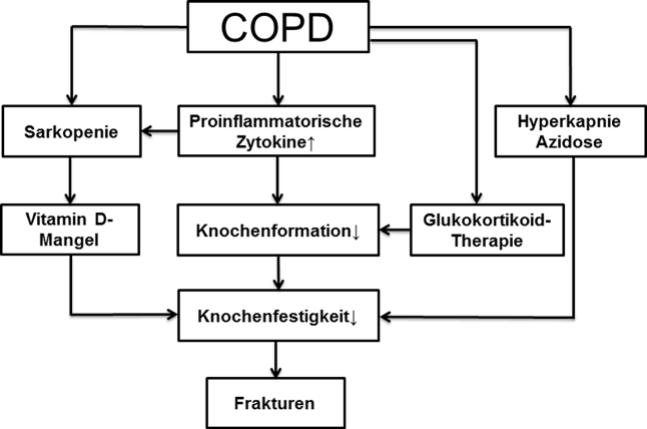

Weitere pathogenetische Mechanismen könnten die Hyperkapnie, die Azidose und der Vitamin D‑Mangel darstellen (Abb. 2; [25–27]). Letztlich muss auch aus klinischer Sicht bedacht werden, dass die oben beschriebenen krankheitsspezifischen Mechanismen sich zu den allgemeinen Osteoporose-Risikofaktoren (wie Alter, Geschlecht) addieren.

## Effekte von Glukokortikoiden auf den Knochenstoffwechsel

**Figure 3:**
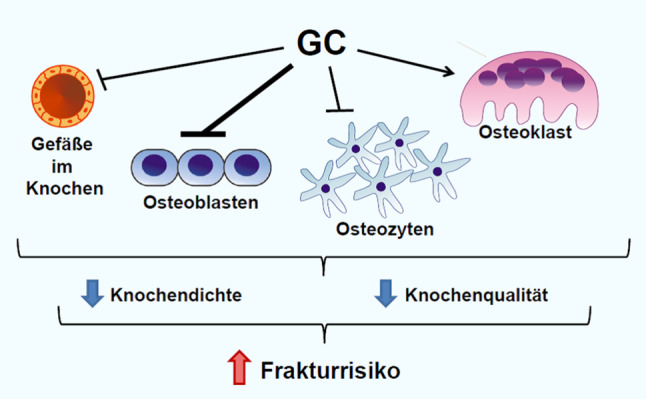

Die systemische Verabreichung von Glukokortikoiden führt unumstritten dauer- und dosisabhängig zur einer verminderten Knochenmineraldichte sowie zu einem erhöhten Frakturrisiko [28]. Dies wurde in zahlreichen Studien mit chronisch-entzündlichen Erkrankungen dokumentiert, darunter auch Studien, die explizit Patientenkollektive mit COPD untersucht haben. Eine Meta-Analyse von 5 Studien mit insgesamt knapp 1800 PatientInnen zeigte, dass PatientInnen mit COPD, die länger als 4 Monate OCS einnahmen, ein 2,3-fach erhöhtes Risiko für vertebrale Frakturen aufweisen [29]. Das erhöhte Frakturrisiko ist dabei zumindest teilweise unabhängig von der KMD, da PatientInnen, die Glukokortikoide einnehmen trotz gleicher KMD gemessen mit DXA ein erhöhtes Frakturrisiko haben, als PatientInnen die keine Glukokortikoide einnehmen [30]. Die Abnahme der KMD sowie das erhöhte Frakturrisiko entwickeln sich rasch, bereits nach 3–6 Monaten Glukokortikoidtherapie, und normalisieren sich wieder nach Absetzen der Therapie. Auf zellulärer Ebene unterdrücken Glukokortikoide potent die Osteoblastendifferenzierung und ihre Funktion, welches zu einer starken Hemmung der Knochenformation führt (Abb. 3;[31–33]). Osteoklasten werden hingegen stimuliert, obwohl die Datenlage hier teilweise kontrovers diskutiert wird [34]. Darüber hinaus führen Glukokortikoide zur vermehrten Apoptose von Osteozyten sowie zu einer Abnahme der Knochenvaskularisierung und Knochenhydration, welches mit eingeschränkten Knochenmatrixeigenschaften einhergeht (Abb. 2; [31, 35]).

Während die Datenlage bei den systemisch verabreichten Glukokortikoiden recht klar ist, werden die Effekte von ICS auf den Knochen bei PatientInnen mit pneumologischen Erkrankungen noch kontrovers diskutiert.

Zwei systematische Meta-Analysen zur COPD wurden veröffentlicht, die beide keinen signifikanten Einfluss von ICS auf das Frakturrisiko gezeigt haben [36, 37]. Differenziert man jedoch genauer nach Therapiedauer und Dosis, zeigen weitere Studien bzw. Meta-Analysen kleine, aber signifikante Anstiege im Frakturrisiko. So fanden Loke et al. ein erhöhtes Frakturrisiko bei PatientInnen mit COPD, welches dosisabhängig je 500 µg Beclomethason oder Äquivalent um 9 % ansteigt [38]. Die „Lung Health Study Research Group“ sowie Scanlon et al. fanden darüber hinaus, dass hohe ICS Dosen mit einem signifikanten Verlust von KMD einhergehen, jedoch erst nach ca. 3 Jahren [39, 40]. Das erhöhte Frakturrisiko, wie auch in der Studie von Goncalves et al. beschrieben, scheint, wie bei OCS, auch unabhängig von der KMD zu sein [41].

Eine ähnliche Datenlage wie zur COPD findet sich auch bei Erwachsenen mit Asthma. Auch hier scheint die Dauer der Therapie sowie die Dosis eine wichtige Rolle zu spielen. Bei höheren Konzentrationen und langanhaltender Therapie sind auch PatientInnen mit Asthma unter ICS einem erhöhten Frakturrisiko ausgesetzt [42]. Die Dosis- und Zeitabhängigkeit zeigt sich auch in den Studien, in denen PatientInnen mit COPD und Asthma gemeinsam untersucht wurden.

Bei Kindern mit Asthma führt die Einnahme von ICS zu einem langsameren Wachstum. Diese Daten sind relativ konsistent und zeigen, dass dieser Effekt vor allem bei Kindern unter 6 Jahren ausgeprägt ist, wohingegen er später (zwischen 6 und 12 Jahren) nicht mehr so ausgeprägt ist, und dass die Hemmung des Wachstums vor allem innerhalb des ersten Jahres der Behandlung stattfindet [42–44].

Insgesamt bleibt festzuhalten, dass systemisch verabreichte Glukokortikoide mit einem erhöhten Frakturrisiko einhergehen. ICS hingegen sind im Erwachsenenalter jedoch nur in hohen Dosen und über einen längeren Zeitraum verabreicht mit einem leicht erhöhten Frakturrisiko verbunden. Weitere prospektive, kontrollierte Studien wären hilfreich, um den Einfluss von ICS auf den Knochen abschließend zu klären.

Trotz der inkonsistenten Datenlage zu Frakturen bei Erwachsenen mit Asthma und COPD besitzen Glukokortikoide viele negative Effekte auf den Knochenstoffwechsel. Aufgrund der klaren Evidenz aus klinischen Studien die zeigen, dass OCS das Frakturrisiko erhöhen, zählen Glukokortikoide (orale und hohe Dosen von inhalativen Glukokortikoiden) zu den Risikofaktoren, die in der Osteoporose-Leitlinie des Dachverbands Osteologie sowie der Frakturrisikoberechnung mit FRAX berücksichtig werden. Auch das American College of Rheumatology hat eine Empfehlung zur Prävention und Therapie der Glukokortikoid-induzierten Osteoporose herausgebracht, die Ärzte bei der Behandlung ihrer PatientInnen behilflich sein kann [45].

## Frakturrisiko basierend auf COPD und Nikotinabusus

Rauchen ist bei Frauen und Männern ein unabhängiger mäßiger Risikofaktor für Wirbelkörperfrakturen, Hüftfrakturen und periphere Frakturen [46, 47]. In einer Metaanalyse von 10 prospektiven Kohortenstudien mit > 350.000 eingeschlossenen Teilnehmerinnen mit einem Alter von 20 bis 93 Jahren zeigt sich eine statistisch signifikante Assoziation mit Hüftfraktur für derzeitige Raucherinnen gegenüber Nicht-Raucherinnen (relatives Risiko, RR 1,30). Mehr als 15 Zigaretten/Tag erhöhen signifikant das Frakturrisiko (RR 1,26). In drei Studien, die das RR für Frakturen bei Frauen, die derzeit rauchen gegenüber Nichtraucherinnen untersuchte, zeigt sich bei den derzeitigen Raucherinnen ein signifikant höheres Risiko für eine Hüftfraktur (RR 1,54). Es zeigt sich allerdings kein statistisch signifikant erhöhtes Risiko für ehemalige Raucherinnen gegenüber Nicht-Raucherinnen (RR 1,02) und ehemaligen Raucherinnen gegenüber derzeitigen Raucherinnen bis 9 Jahre, ab dem 10. Jahr wird das Risiko geringer (Rauchen aufgehört < 5 Jahre RR 1,01; Ende seit 5–9 Jahren RR 1,10; und Rauchen beendet für 10 Jahre RR 0,70) [48, 49].

Die COPD ist bei Frauen und Männern ebenfalls ein mäßiger Risikofaktor für osteoporotische Frakturen und Hüftfrakturen (RR 1,3–1,6 für Frauen, 1,1–1,4 für Männer) [50, 51]. Daten aus einer schwedischen Studie zeigen, dass die COPD bei Männern ein mäßiger bis starker Risikofaktor für vertebrale Frakturen ist (RR 1,67–4,4).[52] Als besonderer Risikofaktor vertebraler Frakturen erscheinen ein niedriger BMI (median 21 kg/m^2^) und COPD (OR 11,0) zu sein [53]. In einer Meta-Analyse von 13 fallkontrollierten Studien war das Risiko für eine weitere Schenkelhalsfraktur nach stattgehabter Schenkelhalsfraktur höher bei bestehender Lungenerkrankung (OR 2,58) [54].

## Frakturrisiko bei Lungentransplantation

Die Funktionsverschlechterung lebenswichtiger Organe führt im Rahmen des Krankheitsverlaufes zu Knochenabbau mit erhöhtem Frakturrisiko [55]. Eine Organtransplantation verbessert signifikant die Lebenserwartung und die Lebensqualität des PatientInnen, die Knochenqualität bleibt allerdings reduziert oder nimmt, vor allem im ersten Jahr nach Transplantation, noch weiter ab und es kommt dadurch zur Steigerung des Frakturrisikos. Am Beispiel von lungentransplantierten Frauen und Männern fand sich in der 12-monatigen Phase vor Transplantation bereits eine Prävalenz für Wirbelkörperfrakturen von 18 %. In der 6–18 monatigen Nachbeobachtungsphase erhöhte sich diese Prävalenz auf über 50 % [56]. Mehr als 60 % der PatientInnen mit end-stage Lungenerkrankungen haben eine herabgesetzte Knochendichte [57, 58]. Risikofaktoren für einen Knochenverlust vor der Transplantation sind Alter, die Diagnose COPD, intermittierende oder kontinuierliche Steroidtherapie, Nikotinabusus, sowie Verlust an Körpergewicht und Muskelmasse (pulmonale Kachexie) durch zunehmende körperliche Dekonditionierung [59].

Aufgrund dieser hohen Inzidenz einer Osteoporose ist im Rahmen der Evaluation zur Lungentransplantation auch eine Erhebung der Knochendichte und des Knochenstoffwechsels erforderlich und bei erhöhtem Frakturrisiko sollte eine entsprechende medikamentöse und physikalische Therapie eingeleitet werden [59]. Eine hochgradige Osteoporose mit oder ohne Wirbelkörperfrakturen stellt eine relative Kontraindikation für eine Transplantation dar. Die Inzidenz solcher Frakturen liegt bei > 30 % aller PatientInnen auf der Transplant-Warteliste [60]. Darüber hinaus kommt es innerhalb von 6–12 Monaten nach Lungentransplantation zu einer signifikanten Abnahme der Knochendichte (> 5 %) trotz Vitamin D und Kalzium Substitution. Dadurch ist die Inzidenz osteoporotischer Frakturen deutlich erhöht (zwischen 15–37 %) [61, 62].

Zwei randomisierte kontrollierte Studien zeigten eine positive Auswirkung von Krafttraining nach Lungentransplantation auf die Knochendichte bereits nach 6 Monaten, und eine Kombination mit einer Alendronat-Therapie führte zu einer zusätzlichen Verbesserung [63, 64]. Weitere Studien konnten einen positiven Effekt auf die KMD unter antiresorptiver Therapie oder Hormonersatztherapie zeigen [65]. Bislang gibt es noch keine Studien über den Effekt von Denosumab, Teriparatid oder selektiven Östrogenrezeptormodulatoren auf den Knochenstoffwechsel bei PatientInnen nach Lungentransplantation. Der Einsatz von Denosumab bei PatientInnen nach Leber‑, Pankreas- und Nierentransplantation zeigte eine signifikante Verbesserung der Knochenmineraldichte und adäquate Hemmung der erhöhten Knochenstoffwechselparameter, ohne Hinweis auf eine mögliche Erhöhung der Inzidenz von Atemwegsinfekten unter Therapie [66].

Diese Daten unterstreichen jedoch, dass jeder Lungentransplantations-Kandidat, der auf die Warteliste kommt, bei entsprechend reduzierter KMD und erhöhtem Frakturrisiko mit einer spezifischen Osteoporosetherapie behandelt werden sollte und darüber hinaus ein regelmäßiges Monitoring des Therapieerfolges indiziert ist [66]. (Vgl. Kapitel Therapieoptionen der Osteoporose).

## Anamnese und Erhebung von Risikofaktoren bei chronischen pneumologischen Erkrankungen

Die Anamnese und Erhebung von klinischen Risikofaktoren stellt einen wichtigen Pfeiler in der Identifikation von PatientInnen mit erhöhtem Frakturrisiko dar. Neben allgemeinen Risikofaktoren, Familienanamnese (insbesondere Hüftfraktur der Eltern), Lebensstilfaktoren wie Nikotin- und Alkoholkonsum, Ernährungs- und Medikamentenanamnese, körperliche Aktivität und Sturzneigung stellen Untergewicht und Sarkopenie ein erhöhtes Risiko dar.

Die Diagnose einer chronischen pneumologischen Erkrankung wie Asthma und COPD stellt gemäß österreichischer Leitlinie und DVO Leitlinie einen unabhängigen Risikofaktor für Osteoporose dar. Das Vorliegen vertebraler Frakturen, niedriger Knochendichte, Osteoporose und Vitamin D‑Mangel wurden in zahlreichen Studien als Risikofaktoren für vermehrte Hospitalisierungen bei COPD PatientInnen identifiziert [67–69]. Darüber hinaus sind vertebrale Frakturen bei COPD PatientInnen mit einer erhöhten 2‑Jahres-Mortalität assoziiert und jede vertebrale Fraktur reduziert die forcierte Vitalkapazität um 9 % [69, 70].

PatientInnen mit chronischen pneumologischen Erkrankungen, insbesondere COPD, leiden häufig an einem Verlust von Muskelmasse, wodurch das Risiko für Stürze und einen Verlust an Knochenmasse steigt. Sarkopenie wird mit einer Prävalenz von 15–25 % bei COPD PatientInnen in der Literatur angegeben und stellt damit eine häufige extrapulmonale Manifestation dar [71]. Darüber hinaus besteht ein Zusammenhang zwischen Untergewicht bei COPD PatientInnen und einer herabgesetzten Überlebenswahrscheinlichkeit [72]. Eine Unterstützung der COPD PatientInnen bei der Optimierung der Ernährung und eine damit verbundene Gewichtszunahme wirkt sich nachweislich positiv auf die Prognose aus, jedoch ohne nachweisbarer Zunahme der Lungenfunktion [73]. Daher ist eine ausreichende alimentäre Proteinzufuhr ebenso entscheidend wie ein muskuläres Aufbautraining. Als weiterer Risikofaktor ist speziell bei PatientInnen mit chronischen pulmologischen Erkrankungen eine systemische aber auch hochdosierte inhalative Glukokortikoidtherapie zu berücksichtigen, da das Risiko für Osteoporose und Frakturen erhöht ist. (Vgl. Kapitel Effekte von Glukokortikoide auf den Knochenstoffwechsel) [74–76].

### Niedrig-traumatische Frakturen

Sind bereits niedrig-traumatische Frakturen, insbesondere Wirbelkörper‑, Hüft‑, Oberarm- und Radiusfrakturen, aufgetreten stellt dies einen entscheidenden Risikofaktor dar. Das klinische Bild der manifesten Osteoporose mit Frakturen und deren assoziierten Folgen (Schmerz, Deformierung und Funktionseinschränkung) führt zu einer deutlichen Einschränkung der Lebensqualität. Die typische, niedrig-traumatische Fragilitätsfraktur der Osteoporose ist durch ein Trauma gekennzeichnet, das üblicherweise nicht für eine Fraktur eines gesunden Knochens ausreicht, wie z. B. ein Sturz aus dem Stand oder geringer Höhe oder ohne größere Krafteinwirkung [49, 77]. Eine Auflistung allgemeiner, klinischer und medikamentöser Risikofaktoren, die in die Beurteilung des Frakturrisikos mit einfließen sind in der österreichischen Leitlinie zusammengefasst [77]. Darüber hinaus findet sich bei einer Vielzahl an chronischen oder malignen Erkrankungen eine relative Risikoerhöhung [77].

## Bildgebende Verfahren

Eine Beurteilung des Frakturrisikos unter ausschließlicher Verwendung der DXA-basierten KMD würde mit einer hohen Spezifität, jedoch niedriger Sensitivität einher gehen und den additiven Effekt der klinischen Risikofaktoren verpassen [77]. Daher ist eine Kombination aus klinischen Risikofaktoren und KMD-Messung empfohlen, um das Frakturrisiko abzuschätzen.

### DXA Knochendichtemessung

Die Bestimmung der KMD mittels DXA (Dual-Energy X‑Ray Absorptiometry, deutsch: 2‑Spektren Röntgen-Absorptiometrie) stellt den Goldstandard in der bildgebenden Diagnostik in der klinischen Routine dar. Die empfohlenen Messorte sind Lendenwirbelsäule, Femurhals und Gesamtfemur. Eine erniedrigte KMD am proximalen Femur und/oder Lendenwirbelsäule geht mit einem gesteigerten Risiko für Osteoporose-assoziierte Frakturen bei Frauen und Männern einher (DVO A). Eine Basisdiagnostik inkl. DXA wird bei Fragilitätsfrakturen ab einem Alter von 50 Jahren, nach 3 Monaten systemischer Glukokortikoidtherapie ≥ 7,5 mg/d Prednisolonäquivalent oder bei bestehendem Risikoprofil für osteoporotische Frakturen bei Frauen ab 50 Jahren und Männern ab 60 Jahren empfohlen.

### Trabecular Bone Score (TBS)

TBS ist ein Texturparameter, welcher ergänzende Informationen über die Knochenmikroarchitektur aus einer bereits existierenden DXA Untersuchung der Lendenwirbelsäule liefert. Die Software analysiert die unterschiedlichen Grau-Werte der zugrundeliegenden Pixel innerhalb der Wirbelkörper und berechnet einen entsprechenden einheitlosen Wert. Ein TBS > 1,350 spricht für einen Normalbefund. Je niedriger der TBS, desto ausgeprägter das trabekuläre Strukturdefizit. Die Analyse erfolgt bei vorhandener Software unmittelbar während einer Knochendichtemessung, kann jedoch auch bestehende Messungen retrospektiv auswerten. Dadurch ergibt sich keine zusätzliche Strahlenbelastung für die PatientInnen und degenerative osteoproliferative Veränderungen beeinflussen die TBS Auswertung nicht [78]. In mehreren prospektiven Studien konnte ein erniedrigter TBS unabhängig von der Knochenmineraldichte zur Vorhersage des Frakturrisikos beitragen (DVO A) und multiple lineare und logistische Regressionsmodelle zeigten unter anderen eine Assoziation eines herabgesetzten TBS mit den Risikofaktoren systemische Glukokortikoidtherapie, prävalente Osteoporose-assoziierte Fraktur und COPD [79]. Die Frakturrisikoberechnung mittels FRAX bietet die Möglichkeit einer TBS-Korrektur, was insbesondere von Vorteil ist, wenn die Knochendichte nicht allzu sehr reduziert ist, jedoch ein trabekuläres Strukturdefizit vorliegt. (Vgl. Kapitel Therapieentscheidung und -ziele).

### Knochenmikroarchitektur bei COPD PatientInnen

Bei COPD PatientInnen mit Wirbelkörperfrakturen konnten TBS und KMD als unabhängige Risikofaktoren identifiziert werden. Darüber hinaus besteht ein Zusammenhang von systemischer Inflammation und einer Reduktion der trabekulären Struktur gemessen mittels TBS, während ein Verlust von KMD mit Gewichtsverlust assoziiert ist [80].

Die hochauflösende periphere quantitative Computertomographie (HR-pQCT) erlaubt eine in-vivo Diagnostik der Knochenmikroarchitektur mit einer Auflösung von 82 µm, welche bei Geräten der jüngsten Generation sogar noch unterschritten wird. Darüber hinaus ist über Finite-Element-Analysen eine Beurteilung der Festigkeit des Knochens möglich. Diese Methode stellt keine Routinediagnostik dar, liefert jedoch interessante Informationen über Mikroarchitekturstörungen bei unterschiedlichen Erkrankungen. So fand sich bei männlichen Patienten mit Osteoporose eine rarefizierte Knochenstruktur und Festigkeit unabhängig von der Diagnose einer COPD, während bei Patienten mit normaler KMD die Diagnose einer COPD mit einer herabgesetzten kortikalen Dicke einherging [81]. Die akkurateste Beurteilung der Knochenstruktur und Knochenqualität kann über eine transiliakale Knochenbiopsie erzielt werden. In einer Studie wurden postmenopausale Frauen mit COPD, herabgesetzter KMD, jedoch ohne den Risikofaktor einer systemischen Glukokortikoidtherapie biopsiert und es fand sich neben einem erniedrigten trabekulären Knochenvolumen und einer rarefizierten trabekulären Struktur auch kortikale Strukturdefekte. Darüber hinaus konnte eine Korrelation der Knochenmikroarchitekturstörung mit der Dauer und Intensität des Nikotinkonsums nachgewiesen werden [18].

### Radiologische Diagnostik

Im Rahmen der Abklärung empfiehlt sich aus osteologischer Sicht eine Bildgebung der Wirbelsäule bei Verdacht bzw. zum Ausschluss von Wirbelkörperfrakturen. Neben akuten oder chronischen Rückenschmerzen können vertebrale Frakturen aber auch klinisch nicht evident sein. Daher ist insbesondere bei hohem Lebensalter, signifikantem Größenverlust oder Abnahme des Rippen-Becken-Abstands, einer herabgesetzten KMD oder vorangegangenen nicht-vertebralen Frakturen eine konventionelle Röntgendiagnostik von Brust- und Lendenwirbelsäule anzustreben. Darüber hinaus ist eine Mitbeurteilung der Wirbelkörper von bereits vorliegenden Thorax-Seitaufnahmen oder CT-Untersuchungen sinnvoll [82].

## Labordiagnostik

Mit Hilfe eines Basislabors können mögliche sekundäre Osteoporose-Ursachen ebenso wie Kontraindikationen für potenzielle medikamentöse Therapien identifiziert werden. Hierbei empfiehlt sich die Bestimmung von Nierenfunktions- und Leberfunktionsparameter, C‑reaktives Protein und Blutsenkungsgeschwindigkeit, Blutbild sowie Schilddrüsenparameter.

Zur detaillierteren Beurteilung des Knochens ist eine Erhebung von Kalzium, Phosphat, Parathormon und 25(OH)-Vitamin D3 essentiell, um Störungen des Kalzium-Phosphat-Stoffwechsels zu identifizieren und einen Mangel an Vitamin D mit sekundärem Hyperparathyreoidismus zu identifizieren.

Eine spezifische osteologische Diagnostik stellt die Bestimmung von Knochenformations- bzw. -resorptionsmarker dar wie z. B. CTX (Resorption), Alkalische Phosphatase (AP, Formation) und Osteocalcin (OC; Formation) dar. Als zuverlässiger Routinemarker der Knochenformation hat sich PINP erwiesen. Knochenstoffwechselparameter sind für eine detailliertere Evaluierung hilfreich und sowohl vor Etablierung einer Osteoporosetherapie als auch als Verlaufsparameter sinnvoll einzusetzen. Eine Erhöhung der AP sollte immer unter Berücksichtigung weiterer Leberfunktionsparameter ebenso wie Calcium, Phosphat und Vitamin D erfolgen. Eine Erhöhung kann neben einer Osteomalazie auch für seltene Knochenerkrankungen wie z. B. M. Paget oder sekundär-blastomatöse Absiedlungen im Knochen sprechen. Mit Hilfe der Serumelektrophorese können Paraproteinämien von Monoklonaler Gammopathie unklarer Signifikant (MGUS) bis Plasmozytom, aber auch chronisch systemische Inflammation nachgewiesen werden.

## Therapieentscheidung und -ziele

Die Häufigkeit der Osteoporose nimmt mit dem Alter zu. Pneumologische Erkrankungen stellen einen klinischen Risikofaktor für Fragilitätsfrakturen dar. Eine medikamentöse Therapie sollte dann in Betracht gezogen werden, wenn in der Zusammenschau ein Behandlungsvorteil zu erwarten ist. Die Entscheidung für eine knochenspezifische Therapie basiert auf der Klinik und dem individuellen Risiko [83].

**Figure 4:**
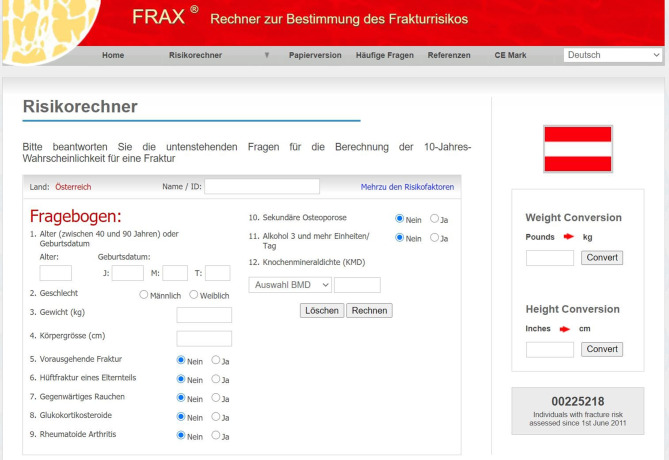

Im zertifizierten Web-basierten FRAX-Risikorechner werden für Patientinnen und Patienten im Alter zwischen 40 und 90 Jahren entsprechend den hinterlegten österreichischen Frakturdaten dichotomisiert elf Fragen erhoben. Die zwölfte Frage zur KMD des Schenkelhalses ist fakultativ. Ein 10-Jahres-Frakturrisiko für alle major osteoporotic fractures (MOF) und für eine Hüftfraktur wird individuell berechnet (Abb. 4; [84]).

Zu den MOF zählen Fragilitätsfrakturen im Erwachsenenalter:

- spontan oder als Folge eines (leichten) Traumas aufgetreten
- bei einem gesunden Menschen hätte das Ereignis nicht zu einer Fraktur geführt
- Anatomische Lokalisation: Hüfte, Wirbelkörper, Humerus, Handgelenk (Radius, Ulna), Becken, (multiple) Rippen

Ein 10-Jahres-Frakturrisiko von ≥20 % für MOF und ≥ 5 % für Femurfrakturen wird in Österreich als sinnvolle Therapieschwelle betrachtet. Daher sollte bei einem individuellen Risiko über einem dieser beiden Schwellenwerte bereits vor der ersten osteoporotischen Fraktur im Sinne einer Prävention eine Osteoporose-spezifische Therapie initiiert werden. Eine niedrig-traumatische Fraktur indiziert in jedem Fall eine osteologische Behandlung.

Eine rein KMD-basierte Entscheidung kann auf Grundlage des DVO Modells erfolgen: Die Therapieschwelle wird fakultativ entsprechend den einbezogenen klinischen Risikofaktoren, soweit diese vorliegen, angehoben. Entscheidungsgrundlage ist der niedrigste Wert der drei Messorte Lendenwirbelsäule (Mittelwert der verwertbaren T‑Scores von L1 bis L4), Schenkelhals und gesamter Femur (Tab. 2; [49, 77]).

**Table 2:** 

Lebensalter in Jahren		T‑Score (Nur anwendbar auf DXA-Werte. Die Wirksamkeit einer medikamentösen Therapie ist für nichtvertebrale Frakturen bei einem T‑Score > −2,0 nicht sicher belegt.)				
Frau	Mann	−2,0 bis −2,5	−2,5 bis −3,0	−3,0 bis −3,5	−3,5 bis −4,0	< −4,0
50–60	60–70	*Nein*	*Nein*	*Nein*	*Nein*	Ja
60–65	70–75	*Nein*	*Nein*	*Nein*	Ja	Ja
65–70	75–80	*Nein*	*Nein*	Ja	Ja	Ja
70–75	80–85	*Nein*	Ja	Ja	Ja	Ja
> 75	> 85	Ja	Ja	Ja	Ja	Ja

Die Anhebung der Therapiegrenze sollte für alle genannten Risiken allein oder in Kombination nur bis zu einem maximalen T‑Score von −2,0 erfolgen. In der Regel sollten nicht mehr als zwei Risikofaktoren additiv bei der Anhebung der Therapiegrenze berücksichtigt werden.

Anpassung um +1,0 bei pneumologischen Erkrankungen:

- OCS ≥ 2,5 mg und < 7,5 mg Prednisolonäquivalent täglich für mehr als drei Monate
- Drei und mehr niedrigtraumatische Frakturen in den letzten 10 Jahren (Ausnahme: Finger‑, Zehen‑, Schädel- und Knöchelfrakturen)

Anpassung um +0,5 bei pneumologischen Erkrankungen:

- Singuläre Wirbelkörperfraktur 1. Grades; Nichtvertebrale Frakturen > 50. Lebensjahr (Ausnahme: Finger‑, Zehen‑, Schädel- und Knöchelfrakturen)
- Proximale Femurfraktur bei Vater oder Mutter
- Multiple intrinsische Stürze
- Immobilität
- Rauchen, COPD, und/oder hohe Dosen ICS
- Protonenpumpeninhibitoren bei chronischer Einnahme
- Erhöhung des hochsensitiven C‑reaktiven Proteins (hsCRP)

**Figure 5:**
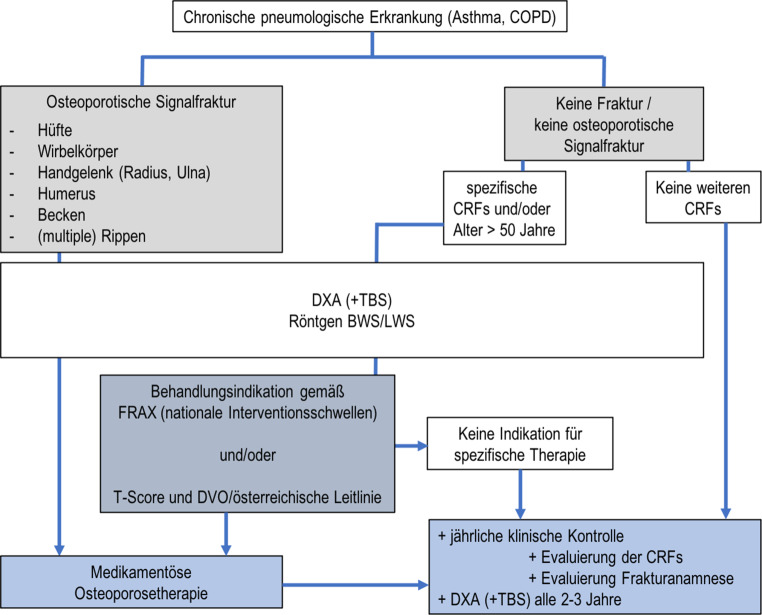

Bei niedrigtraumatischen Osteoporose-typischen Frakturen in der Anamnese ist immer eine spezifische Osteoporosetherapie über Vitamin D/Kalzium hinaus indiziert. Die Therapie kann in diesen Fällen auch ohne Messung der Knochenmineraldichte begonnen werden. In Abb. 5 ist ein Algorithmus zur Bestimmung des Frakturrisikos und der Interventionsschwelle bei PatientInnen mit chronischen pulmologischen Erkrankungen zusammengefasst.

## Vitamin D bei Lungenerkrankungen

Ein Vitamin D‑Mangel ist sowohl in der Allgemeinbevölkerung als auch bei Menschen mit chronischen Erkrankungen häufig [85]. Dieser ist meist definiert als 25OHD im Serum < 20 ng/ml oder ein schwerer Mangel als < 12 ng/ml (bzw. 50/30 nmol/l – Umrechnungsfaktor zu nmol/l: 2,5). Gerade bei Menschen mit chronischen Lungenerkrankungen scheint eine Vitamin D Gabe ineffizienter zu sein als bei Kontrollen, hinweisend auf die Sinnhaftigkeit von Spiegelbestimmungen [86]. Vitamin D ist wichtig für das muskuloskelettale System, aber auch das Immunsystem [87].

Die Einleitung einer osteoprotektiven antiresorptiven Therapie bei Calcium‑/Vitamin D‑Mangel kann zu gefährlichen Komplikationen führen und muss daher vermieden werden. Eine kombinierte Calcium‑/Vitamin D Gabe bzw. alleinige Vitamin D Gabe bei ausreichender nutritiver Calciumzufuhr ist daher bereits vor Therapieeinleitung sinnvoll.

Interventionsstudien belegen, dass Vitamin D Atemwegsinfekte reduzieren kann – vor allem bei täglicher oder wöchentlicher Einnahme (im Gegensatz zu selteneren Intervallen) und bei schwerem Mangel [88, 89]. Dies kann auch für COVID-19 relevant sein. Für COVID-19 selbst gibt es derzeit nur wenige und kleine Interventionsstudien zum Wert einer Vitamin D Supplementierung.

Für COPD und Asthma legen Daten aus randomisiert kontrollierten Studien nahe, dass Vitamin D bei Mangel Exazerbationen und Hospitalisationen reduzieren kann, sowohl bei Kindern als auch bei Erwachsenen [90, 91].

Auch für die Entwicklung eines Diabetes mellitus gibt es Hinweise, besonders klar ist die Datenlage zum Benefit einer Vitamin D Supplementierung jedoch für die Vermeidung von akuten Atemwegsinfekten und die Allgemein- und Krebssterblichkeit, höchst relevante Endpunkte für Lungenkranke mit oder ohne Osteoporose.

Rezent gibt es auch im Zusammenhang von COVID‑19 und Vitamin D interessante neue Daten. Niedrigere Vitamin D‑Spiegel waren mit höheren Raten von Serokonversion bei Menschen aus Gesundheitsberufen assoziiert [92] und hospitalisierte COVID-19 PatientInnen hatten eine höhere Prävalenz einer Vitamin D Defizienz, wobei kein Zusammenhang mit dem Schweregrad der Erkrankung gefunden wurde [93]. Kleine Interventionsstudien (*n* < 250) mit interessanten Ergebnissen wurden ebenfalls rezent publiziert [94–96], wobei Bolusgaben ineffektiv zu sein scheinen [97]. Zu diesem Thema findet sich auf der Website vdmeta.com ein aktualisierter Überblick.

## Therapieoptionen der Osteoporose

### Basisprophylaxe mit Vitamin D und Kalzium

Die Kalzium- und Vitamin D Substitution ist sowohl eine eigenständige Therapiemöglichkeit der Osteoporose, als auch die absolut notwendige Basis jeder spezifischen Osteoporosetherapie.

Eine ausreichende Versorgung mit Vitamin D ist eine wichtige Voraussetzung für die Knochengesundheit. Eine Vitamin D3 Serumkonzentration < 20 ng/ml (50 nmol/l) ist mit einem erhöhten Risiko für proximale Femurfrakturen und nichtvertebralen Frakturen verbunden. Wir empfehlen daher den Vitamin D‑Spiegel bei Menschen mit akuten und chronischen Lungenerkrankungen zu messen und einen Zielspiegel von > 20–30 ng/ml (nicht über 60 ng/ml) anzustreben. Dies gelingt üblicherweise mit Tagesdosen von 800 bis 4000 IU, wöchentliche Gaben sind akzeptabel, länger sollte das Dosierungsintervall jedoch nicht sein. Natives Vitamin D (Cholecalciferol, Vitamin D3; 1 μg Vitamin D3 entspricht 40 IE Vitamin D3) als Arzneimittel ist primär zu empfehlen, da die zahlreichen verfügbaren Nahrungsergänzungsmittel keiner strengen Qualitätskontrolle unterliegen. Die Einnahme soll mit den Mahlzeiten erfolgen, da dies die Resorption verbessert. Im Einzelfall kann bei Malabsorption eine parenterale (intramuskuläre) Gabe von 100.000 IE Cholecalciferol notwendig sein. Die Gabe der aktiven Form von Vitamin D – Calcitriol (1,25-Dihydroxycholecalciferol) ist nur bei schwerer Niereninsuffizienz indiziert.

Eine ausreichende Kalziumzufuhr ist primär über die Nahrung sicherzustellen. Patientinnen und Patienten mit Osteoporose (mit oder ohne spezifischer Osteoporosetherapie) sollen daher täglich 1000 mg Kalzium aufnehmen, vorzugsweise über die Nahrung. Ist dies nicht möglich, sind Kalziumsupplemente erforderlich. Pro Einnahme wird eine Dosis von maximal 500 mg Kalziumsupplement empfohlen [77].

### Spezifische Osteoporosetherapie bei pneumologischen Erkrankungen

Keine einzige randomisierte Studie hatte bisher als Endpunkt die Wirksamkeit einer spezifischen Osteoporosetherapie bei PatientInnen mit einer pneumologischen Erkrankung. Daher basieren die Empfehlungen für das Management dieser PatientInnen mit einem erhöhten Frakturrisiko auf empirischen Daten und der klinischen Erfahrung. Die klinische Evidenz in Bezug auf die Effizienz einer antiresorptiven oder anabolen Osteoporosetherapie bei gleichzeitiger pneumologischer Erkrankung abseits der Onkologie beruht daher auf post hoc Analysen von Subgruppen in großen randomisierten Osteoporose-Studien und auch einer kleinen Anzahl von Observationsstudien [98, 99].

Grundsätzlich sind sämtliche Medikamente zur Behandlung der Osteoporose auch bei Patientinnen und Patienten mit einer pneumologischen Erkrankung möglich und zugelassen. Da sowohl die Grunderkrankung als auch die Osteoporose eine chronische Erkrankung mit einem dauerhaft erhöhten Risiko für sekundäre Komplikationen sind, ist bei einem erhöhten Knochenbruchrisiko die Indikation für eine langfristige Behandlung indiziert.

#### Bisphosphonate

Bisphosphonate (Alendronat, Risedronat, Ibandronat, Zoledronat) sind potente Inhibitoren der Knochenresorption. Sie werden an metabolisch aktiven Umbaueinheiten im Knochen abgelagert und bewirken eine Apoptose von Osteoklasten. Die Resorptionsaktivität wird im Gesamtskelett deutlich gedämpft und das Frakturrisiko reduziert.

Oral werden Bisphosphonate nur in geringem Ausmaß (maximal 3 %) resorbiert; die Einnahme erfolgt stets nüchtern in ausreichendem Abstand zur Nahrungsaufnahme, mit ausreichend Wasser und in aufrechter Körperhaltung, um Irritationen der Ösophagusschleimhaut zu vermeiden.

Bei intravenöser Bisphosphonatgabe kann, überwiegend bei erstmaliger Verabreichung, eine sogenannte „Akutphasereaktion“ – im Wesentlichen ein grippeähnliches Zustandsbild mit Fieber und Muskelschmerzen – auftreten, die in der Regel innerhalb von 36 h nach intravenöser Gabe beginnt und dann 24–48 h anhält.

Bei allen Bisphosphonaten stellen die Hypokalzämie, eine erhebliche Nierenfunktionseinschränkung oder eine Gravidität eine Kontraindikation dar.

Bisphosphonate haben eine lange Verweildauer im Knochen. Residuale Wirkungen auf den Knochenstoffwechsel lassen sich auch nach Beendigung der Bisphosphonattherapie nachweisen. Das Auftreten von atypischen Femurfrakturen ist sehr selten, scheint aber unter einer Langzeitgabe mit Bisphosphonaten zuzunehmen. Kiefernekrosen sind bei dieser für Osteoporose zugelassenen Therapie eine mutmaßlich seltene Nebenwirkung. Eine Kontrolle des Zahnstatus ist allerdings vor Therapiebeginn empfehlenswert.

Es gibt keine durch Frakturdaten validierten individuellen Entscheidungskriterien für die Wiederaufnahme einer Therapie nach einer Therapiepause oder einen weiteren Therapieverzicht in Abhängigkeit von Veränderungen der KMD, der Knochenumbaumarker oder anderer messtechnischer oder klinischer Kriterien. Datenbankanalysen geben allerdings Hinweise auf einen Wiederanstieg des Knochenbruchrisikos nach Absetzen einer Bisphosphonattherapie [77].

#### Denosumab

Denosumab ist ein monoklonaler Antikörper gegen RANKL, der die Reifung und Aktivierung der Osteoklasten hemmt. Es wird alle sechs Monate subkutan verabreicht und wird nicht renal eliminiert.

Bei der Behandlung der postmenopausalen Osteoporose ist eine Reduktion von vertebralen und nichtvertebralen Frakturen inklusive proximaler Femurfrakturen in Studien bis zu 10 Jahre nachgewiesen. Die Wirkung ist unabhängig von einer eventuellen Vorbehandlung mit Bisphosphonaten [100]. Die Behandlungsdauer ist unklar. Nach Absetzen von Denosumab scheint es, im Gegensatz zu den Bisphosphonaten, zu einem raschen Anstieg des Knochenumbaus und in weiterer Folge zu einer Abnahme der KMD sowie einem Anstieg des Risikos für vertebrale Frakturen zu kommen. Kiefernekrosen und atypische Femurfrakturen sind bei dieser für Osteoporose zugelassenen Therapie eine mutmaßlich sehr seltene Nebenwirkung [77].

#### Raloxifen

Raloxifen ist ein selektiver Östrogenrezeptor-Modulator (SERM), der die Knochenresorption hemmt und das Frakturrisiko für vertebrale Frakturen reduziert (nicht für nicht-vertebrale Frakturen und proximale Femurfrakturen). Raloxifen ist zugelassen für die Prävention und für die Therapie der Osteoporose bei postmenopausalen Frauen.

Ein bedeutender zusätzlicher Effekt ist die Reduktion des relativen Risikos eines invasiven (Östrogenrezeptor-positiven) Mammakarzinoms um 79 %. Eine unerwünschte Nebenwirkung ist die Erhöhung des thromboembolischen Risikos [77].

#### Teriparatid

Teriparatid, ein aminoterminales Fragment des Parathormons, wird einmal täglich subkutan über 24 Monate angewandt. Der osteoanabole Effekt beruht auf einer Beschleunigung der Reifung und Stimulierung von Osteoblasten. Die Therapie ist zur Behandlung der schweren postmenopausalen Osteoporose, männlichen und glukokortikoid-induzierten Osteoporose zugelassen. Diese therapeutische Möglichkeit kann PatientInnen derzeit nur einmalig über die vollen 24 Monate verabreicht werden.

Im Anschluss an die anabole Reaktion des Knochens kommt es nach Beendigung der Teriparatid-Therapie wiederum zu einem gesteigerten Knochenabbau, weshalb eine sofortige Anschlussbehandlung mit einem Antiresorptivum (Bisphosphonat, Denosumab, SERM) unbedingt notwendig ist [77].

#### Neue/zukünftige Osteoporose Medikamente

Romosozumab, ein Anti-Sclerostin Antikörper, verbessert die BMD und die Mikroarchitektur auf kortikaler und trabekulärer Ebene. Studiendaten bei postmenopausalen Frauen mit einem erhöhten Knochenbruchrisiko zeigen eine außergewöhnlich starke Zunahme der BMD bei monatlicher Applikation. Diese Therapie ist kontraindiziert bei kardiovaskulären und zerebrovaskulären Ereignissen in der Anamnese. Zusammengefasst könnte dieser Antikörper zukünftig eine neue Behandlungsoption auch bei pneumologischen Erkrankungen in der entsprechenden Indikation darstellen [101].

## Verlaufskontrollen

Osteoporose stellt eine chronische Erkrankung dar und ist damit mit einem dauerhaft erhöhten Frakturrisiko assoziiert. Je nach Behandlungsstrategie ist eine regelmäßige Überprüfung des Therapieerfolges durch klinische Kontrollen, Frakturananmnese und Evaluierung des Risikoprofils erforderlich.

Eine frühzeitige Überprüfung der Therapieindikation und des Frakturrisikos sollte bei Änderung der Risikofaktoren erfolgen. Dies kann z. B. das Auftreten von niedrig-traumatischen Frakturen oder die Etablierung einer medikamentösen Therapie mit negativer Auswirkung auf den Knochenstoffwechsel sein, da dadurch die Therapieschwelle eventuell früher erreicht wird.

Gemäß DVO Leitlinie sind KMD-Messungen zur Abschätzung des Therapieerfolges nur bedingt tauglich. Generell wird bei OsteoporosepatientInnen eine Kontrolle der Knochendichte alle 2–3 Jahre empfohlen, jedoch ist der Zeitabstand auch abhängig von der Therapierelevanz. Wenn ein Absinken von −1,0 SD T‑Score therapierelevant ist, sollte eine erneute Messung nicht vor Ablauf von 2 Jahren erfolgen [49, 77].

## Rehabilitation nach Fraktur bei pneumologischen Erkrankungen

Hier gilt es, die Elemente der orthopädisch-traumatologischen und der pneumologischen Rehabilitation zu kombinieren (Tab. 3).

**Table 3:** 

Orthopädisch-traumatologische Aspekte	Pulmologische Aspekte
Remobilisierung	Ausdauertraining (ggf. bereits ab 3. Woche)
Ggf. Mieder oder funktionelle Orthesen	Ggf. neuromuskuläre Elektrostimulation
Ergonomie	Ernährungsberatung
Isometrische Übungen	Atemtherapie
Krafttraining (allgemein ab ca. 6 Monaten, spezifisch ggf. bereits ab ca. 6 Wochen)	
Beweglichkeit (ab ca. 4 Monaten)	Nikotinkarenz
Sturzprophylaxe	Schulung (Erkrankung, Medikamente)
ADL, Hilfsmittel	ADL-Ökonomisierung

### Orthopädisch-traumatologische Aspekte

#### Wirbelkörperfraktur

Um Komplikationen der Immobilität wie Pneumonie, Dekonditionierung oder Dekubitus zu vermeiden, stehen zu Beginn der orthopädisch-traumatologischen Rehabilitation am Beispiel der konservativ behandelten Wirbelkörperfraktur die multimodale Schmerztherapie, bei unzureichender Stabilität eine Miederversorgung (meist für 8–12 Wochen) sowie die rasche Remobilisierung im Vordergrund [102].

Im Rahmen der Physiotherapie erfolgen neben der Remobilisierung und ergonomischen Instruktionen (z. B. en bloc Drehen) isometrische Stabilisierungsübungen zur Verhinderung einer Kyphosierung sowie ggf. ein stufenweiser Miederabbau [103].

Nach etwa drei bis vier Monaten heilen die meisten Frakturen gemäß radiologischer Kriterien [104]. Ab diesem Zeitpunkt und nach erfolgter wird empfohlen, schmerzadaptiert auch an der Verbesserung der Beweglichkeit der Wirbelsäule zu arbeiten.

Generelles Krafttraining soll ab etwa sechs Monaten nach einer osteoporotischen Wirbelkörperfraktur ausgeübt werden [102].

Um das Risiko für weitere Frakturen zu reduzieren, werden Sensomotoriktraining, Beseitigung von Stolperfallen, sowie das Absetzen von Sturz-begünstigenden Medikamenten empfohlen [103].

Durch den Einsatz von funktionellen Orthesen kommt es zu einer Schmerzlinderung, die Kräftigung der Rückenmuskulatur wird unterstützt und die Durchführung der Aktivitäten des täglichen Lebens (ADL) wird erleichtert [102].

Bei allen PatientInnen, die durch die Fraktur an funktionellen Einschränkungen leiden, wird eine ergotherapeutische Beratung bezüglich der ADL, Hilfsmittelabklärung (z. B. Gehhilfen, Anziehhilfen) sowie ggf. ADL-Training empfohlen. Das Ziel ist die Selbstständigkeit möglichst zu erhalten bzw. wiederherzustellen.

Bei schweren neurologischen Ausfällen kann zusätzlich eine pflegerische Unterstützung indiziert sein [103]. Möglicherweise ist auch eine psychosoziale Intervention erforderlich [103].

#### Schenkelhalsfraktur

Eine aktuelle Übersichtsarbeit berichtet, dass die vorliegenden klinischen Leitlinien nur wenige Informationen zur Rehabilitation nach Frakturen enthalten. Insbesondere sind die Art der rehabilitativen Intervention, Intensität, Häufigkeit und Zeitpunkte nicht genau definiert. Jedenfalls wird eine rasche Mobilisierung ab dem ersten postoperativen Tag, mindestens einmal täglich, empfohlen [105].

Da die Schenkelhalsfraktur auch Ausdruck komplexer Funktionsstörungen ist, gilt es in der Rehabilitation folgende Ziele zu erreichen: Reduktion von Schmerz und Schwellung, (Re-), Aktivierung der Muskulatur, Remobilisierung, Hilfsmittel- und ADL-Abklärung [106, 107]. Hierfür ist ein multimodaler Ansatz nötig und es ergänzen sich medikamentöse, diätetische, bewegungstherapeutische und ergotherapeutische Maßnahmen sowie physikalische Modalitäten [106, 107].

Rehabilitative Maßnahmen wie die Atemphysiotherapie zur Pneumonieprophylaxe, sollten unserer Meinung nach bereits präoperativ erfolgen. Die Belastbarkeit der betroffenen Extremität ist von der Art der operativen Versorgung abhängig. Gewisse Bewegungslimitierungen sind z. B. nach Implantation einer Endoprothese nötig, um eine Luxation zu vermeiden.

Die Bewegungstherapie sollte an die Heilungsphasen des Bindegewebes angepasst werden [107, 108]. Während in der Akutphase (Tage 0–5) Atemphysiotherapie, Entstauung, passiv/assistive Mobilisation bis zur Schmerzgrenze und Stehversuche erfolgen, sollte in der Proliferationsphase (Tage 5–21) an der weiteren Mobilisierung, Koordination, Steigerung des Bewegungsumfanges, Gangschulung, Kräftigung und Sensomotorik gearbeitet werden. In der Konsolidierungsphase (Tage 21–60) stehen Narbenbehandlungen, intensivere Kräftigung, Sensomotorik und Sturzprophylaxe im Vordergrund und in der Umbauphase die medizinische Trainingstherapie.

### Pneumologische Aspekte

Am Beispiel der COPD liegen Rekonditionierung, Edukation, Ernährungstherapie und psychosoziale Unterstützung im Fokus [109, 110].

Die Basis stellt das Ausdauertraining dar, welchem idealerweise ein Belastungstest vorausgeht und gemäß der medizinischen Trainingslehre nach Haber dosiert wird [111]. Ausdauertraining reduziert Belastungsdyspnoe, verbessert die muskuläre Sauerstoffutilisation und verringert die Blut-Laktat-Konzentration sowie die CO2 Produktion, was in geringerer Atemarbeit resultiert [109]. Die Belastung sollte zwischen 60–80 % der maximalen Herzfrequenz liegen bzw. gemäß der subjektiven Dyspnoe entsprechend der Borg Skala. Es sollten mindestens zweimal pro Woche bzw. zehn Minuten pro Einheit angestrebt werden. Falls eine Dauerbelastung nicht möglich ist, kann ein Intervalltraining erfolgen [111]. Sauerstoffsupplementation und/oder nicht-invasive Beatmung während des Trainings können erforderlich sein oder auch dabei helfen, höhere Trainingsintensitäten zu erreichen [109].

Bezüglich der Stabilität der Wirbelsäule ist sehr früh (etwa ab der dritten Woche nach der Wirbelkörperfraktur) ein leichtes Ausdauertraining am Sitzfahrrad oder Gehen ohne Stöcke (und somit ohne Rotationskomponente der Wirbelsäule) möglich.

Intensiveres Training oder Trainingsarten, welche Flexionsbewegungen der Wirbelsäule (z. B. Ergometertraining) beinhalten, sollten erst nach radiologisch verifizierter Frakturheilung (nach etwa drei bis vier Monaten) schmerzadaptiert durchgeführt werden. Ausdauersportarten mit Rotationskomponente der Wirbelsäule (z. B. Nordic Walking) wird ab sechs Monaten empfohlen.

Bei ausgeprägter Belastungsdyspnoe sollte zusätzlich eine neuromuskuläre Elektrostimulation erfolgen [111].

Ergänzend wird Krafttraining empfohlen, welches ebenfalls nach der medizinischen Trainingslehre dosiert wird und zweimal pro Woche stattfinden soll, mit einer Intensität 50–85 % des 1‑Wiederholungsmaximums bzw. 10–15 Wiederholungen [111]. Nach einer osteoporotischen Wirbelkörperfraktur kann mit einem Krafttraining nach sechs Monaten begonnen werden. Ein spezifisches Krafttraining der Kniestrecker und/oder Kniebeuger ohne fortgeleitete Belastung der Wirbelsäule (z. B. mittels leg extension bzw. leg curl nach entsprechender Instruktion und unter Supervision) ist nach erfolgter Ödemresorption (ab circa sechs Wochen) möglich, unter Umständen schmerzabhängig bereits früher.

Ergänzend zum Training ist aufgrund der verschiedenen möglichen metabolischen Veränderungen (z. B. Kachexie oder Adipositas) eine Ernährungsberatung indiziert [109, 111].

Ein weiterer zentraler pneumologischer Aspekt ist die Atemphysiotherapie. Entspannungstechniken sind die Basis für das Atemtraining. Bei abnormer Hustenmechanik, beispielsweise durch Muskelschwäche, kann Sekret durch Lagerung, Atemtechniken wie autogene Drainage oder Huffing, sowie mit apparativen Hilfsmitteln (z. B. PEP oder Flutter) leichter mobilisiert werden. Die Sekretelimination hilft die Atemarbeit zu reduzieren, den Gasaustausch zu verbessern sowie Atelektasen und Infektionen zu verhindern.

Bei reduzierter inspiratorischer Atemmuskelkraft sollte ein inspiratorisches Atemmuskeltraining erfolgen. Ein exspiratorisches Atemmuskeltraining kann insbesondere Fatigue reduzieren. Der dynamischen Überblähung kann durch exspiratorische Stenosetechniken (zB Lippenbremse), Flexions- und Expansionstechniken sowie segmentales Atmen entgegengewirkt werden [109, 111]. Atemtherapeutische Techniken, welche Bewegungen des betroffenen Wirbelsäulensegments beinhalten (z. B. Flexionstechniken), empfehlen wir jedoch erst nach radiologisch verifizierter Frakturheilung anzuwenden (nach etwa drei bis vier Monaten).

Zur pneumologischen Rehabilitation zählen weiters die Nikotinkarenz, ADL-Ökonomisierung (energiesparende Maßnahmen), Schulung bezüglich der Erkrankung (Erkennen von Verschlechterungszeichen, Handhabung von Medikamenten wie z. B. Inhalatoren oder Sauerstoff) sowie psychosoziale Unterstützung [109, 111].

## Conclusion

Die pneumologischen Grunderkrankungen führen zu einer Veränderung der trabekulären und kortikalen Mikroarchitektur des Knochens und zu einer Verminderung von osteologischen Formations- und Resorptionsmarkern. Hyperkapnie, Azidose und Vitamin D‑Mangel können diesen Prozess beschleunigen und somit das individuelle Risiko für osteoporotische Fragilitätsfrakturen erhöhen.

Eine Knochendichtemessung mit einem T‑Score < −2,5 ist ein Schwellenwert zur Diagnose der Osteoporose, die überwiegende Mehrzahl aller osteoporotischen Frakturen tritt bei einem T‑Score von > −2,5 auf. Eine niedrig-traumatische Fraktur in der Anamnese indiziert eine osteologische Therapie.

Neben der Optimierung des Vitamin D‑Spiegels sind sämtliche in Österreich zur Behandlung der Osteoporose zugelassenen antiresorptiv oder anabol wirksamen Medikamente auch bei pneumologischen PatientInnen mit einem erhöhten Knochenbruchrisiko entsprechend der nationalen Erstattungskriterien indiziert.
